# Interactions between Major Bioactive Polyphenols of Sugarcane Top: Effects on Human Neural Stem Cell Differentiation and Astrocytic Maturation

**DOI:** 10.3390/ijms232315120

**Published:** 2022-12-01

**Authors:** Kengo Iwata, Farhana Ferdousi, Yoshinobu Arai, Hiroko Isoda

**Affiliations:** 1 School of Integrative and Global Majors, University of Tsukuba, Tsukuba 305-8572, Japan; 2 Nipoo Co., Ltd., Osaka 574-0062, Japan; 3 Alliance for Research on the Mediterranean and North Africa (ARENA), University of Tsukuba, Tsukuba 305-8572, Japan; 4 AIST—University of Tsukuba Open Innovation Laboratory for Food and Medicinal Resource Engineering (FoodMed-OIL), Tsukuba 305-8572, Japan; 5 Faculty of Life and Environmental Sciences, University of Tsukuba, Tsukuba 305-8572, Japan

**Keywords:** sugarcane top, neural stem cells, caffeoylquinic acid, isoorientin, G1 cell cycle arrest, bHLH transcription factor, astrocytic mitochondrial activity

## Abstract

Sugarcane (*Saccharum officinarum* L.) is a tropical plant grown for sugar production. We recently showed that sugarcane top (ST) ameliorates cognitive decline in a mouse model of accelerated aging via promoting neuronal differentiation and neuronal energy metabolism and extending the length of the astrocytic process in vitro. Since the crude extract consists of multicomponent mixtures, it is crucial to identify bioactive compounds of interest and the affected molecular targets. In the present study, we investigated the bioactivities of major polyphenols of ST, namely 3-*O*-caffeoylquinic acid (3CQA), 5-*O*-caffeoylquinic acid (5CQA), 3-*O*-feruloylquinic acid (3FQA), and Isoorientin (ISO), in human fetal neural stem cells (hNSCs)- an in vitro model system for studying neural development. We found that multiple polyphenols of ST contributed synergistically to stimulate neuronal differentiation of hNSCs and induce mitochondrial activity in immature astrocytes. Mono-CQAs (3CQA and 5CQA) regulated the expression of cyclins related to G1 cell cycle arrest, whereas ISO regulated basic helix-loop-helix transcription factors related to cell fate determination. Additionally, mono-CQAs activated p38 and ISO inactivated GSK3β. In hNSC-derived immature astrocytes, the compounds upregulated mRNA expression of PGC-1α, a master regulator of astrocytic mitochondrial biogenesis. Altogether, our findings suggest that synergistic interactions between major polyphenols of ST contribute to its potential for neuronal differentiation and astrocytic maturation.

## 1. Introduction

Neurodegenerative diseases (NDDs) refer to a clinically heterogeneous group of multi-system disorders characterized by progressive deterioration of neuronal function and loss of neurons, including Alzheimer’s disease (AD), Parkinson’s disease (PD), and Huntington’s disease (HD). The major challenge in treating NDDs is that neuronal cell damages are irreversible and non-repairable [1]. Though certain environmental and lifestyle factors combined with genetic factors are implicated in the pathogenesis of NDDs, aging remains the primary risk factor. Cellular changes in the aged brain include mitochondrial malfunction, increased oxidative stress and lipid peroxidation, and damaged nucleus [2], eventually leading to neuronal atrophy and death, abnormal neural signaling, and impaired neurogenesis [2,3].

In addition, neural dysfunction and degeneration can trigger the defensive program known as reactive astrogliosis, which appears as hypertrophy and local proliferation of astrocytes. Reactive astrogliosis functionally provides for neuroprotection, preserving brain homeostasis [4,5]. However, reactive astrogliosis in the aging or degenerating brain is often accompanied by many ‘malignant’ reactive astrocytes that are highly neurotoxic and display disrupted functions, including decreased phagocytic capacity and impaired synaptogenesis [6,7]. Recent studies have also indicated that morphological deficits in astrocytes preceding astrogliosis may contribute to the progression of NDDs [8,9]. Although the biological effects of naturally occurring compounds are well documented, several factors limit the successful clinical implementation of these molecules, including inefficient extraction procedures, difficulties in standardizing functional components, low bioavailability, and toxic effects [10,11].

Accumulating evidence suggests that dietary phytochemicals are beneficial in preventing or delaying brain aging and neurodegenerative diseases. Several plant-derived compounds, such as curcumin, resveratrol, blueberry polyphenols, and citrus flavonoids, have been reported to promote neurogenesis and improve hippocampal function in the adult brain [12,13]. The benefits of these natural products for brain health are attributed to their effects on reducing oxidative stress, enhancing cell signaling, and positively modulating growth factors [14]. Interestingly, some plant-derived molecules have been shown to directly stimulate neural stem cell (NSCs) differentiation in several in vitro culture systems [15,16,17,18].

We have recently reported that oral administration of sugarcane top (ST) ethanolic extract (STEE) could ameliorate spatial learning and memory deficits in senescence-accelerated prone mouse 8 (SAMP8) that progressively develop aging phenotype. STEE improved cortical neurotransmission and stimulated hippocampal neurogenesis [19]. In vivo omics analysis and in vitro experiments with cultured cells have suggested the activation of pathways related to energy metabolism and neurodevelopment is the main mechanism underlying the above effects. Furthermore, a possible role of STEE in the promotion of astrocytic process formation has been suggested [19]. However, since the crude STEE consists of multicomponent mixtures, it is critical to identify the bioactive compounds of interest and elucidate their molecular modes of action to standardize the extract or scale-up of the bioactive lead compounds. In our previous study, HPLC analysis revealed four major polyphenolic constituents in the STEE- 3-*O*-caffeoylquinic acid (3CQA), 5-*O*-caffeoylquinic acid (5CQA), 3-*O*-feruloylquinic acid (3FQA), and Isoorientin (ISO, chemically luteolin-6-C-glucoside). Several in vitro studies showed that 5CQA ameliorated amyloid-β (Aβ)-induced neurotoxicity and promoted neurite outgrowth in rat hippocampal neuronal cells [20,21]. ISO also showed a protective effect against Aβ-induced neuronal damage [22]. However, their effects on NSCs differentiation and developmental progression have not yet been examined. In this context, we ask how the STEE polyphenols contribute to its multifaceted neuroprotective effects.

The aim of this study was to unravel the bioactivities of the major polyphenols of STEE that contribute to its effects on neuronal differentiation and astrocyte morphological maturation. We used human embryonic NSCs (hNSCs) to investigate the molecular mechanism underlying the pro-neurogenic potential of STEE and its polyphenolic constituents, both independently and in combination. We further evaluated their effects on astrocytic mitochondrial activity in hNSC-derived immature astrocytes. In addition, we examined the permeability of the compounds into the brain parenchyma employing an in vitro blood-brain barrier (BBB) model.

## 2. Results

### 2.1. STEE and Its Major Compounds Enhanced Tubulin-β3 Expression in Differentiating hNSCs

hNSCs were cultured with STEE and its compounds to investigate their proneurogenic effect. There were ten different combinations of the compounds. The treatment group of combinations is presented in Table 1.

**Figure 1:**
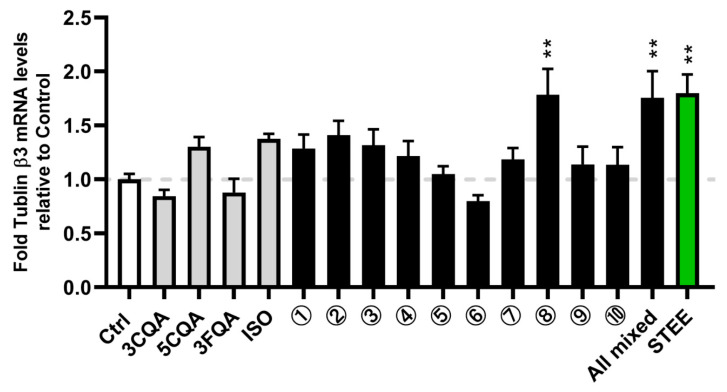
STEE and its major polyphenolic constituents enhanced neuronal marker expression in hNSCs. Cells were treated with STEE and its major polyphenols, 3CQA, 5CQA, 3FQA, or ISO, for 24 h. RNA was isolated from the cells, and tubulin-β3 mRNA (*TUBB3*) levels were measured by qRT-PCR. The relative values are the mean ± SEM (n = 3–4) compared with the control. One-way ANOVA with Dunnett’s post hoc test for the comparisons: ** *p* < 0.01. The description of the number for the treatment groups is indicated in Table 1.

Neuronal marker tubulin-β3 mRNA (*TUBB3*) levels were measured. After incubation with STEE for 24 h, *TUBB3* expression was significantly increased (*p* < 0.01, approximately 1.8-fold) in differentiating hNSCs (Figure 1). The treatment of the four compounds mixture (All mixed) and group 8 also significantly increased the *TUBB3* expression compared with the control (*p* < 0.01). The single treatment of each compound or other combinations had no significant effect (Figure 1). The data suggest that 3CQA, 5CQA, and ISO are responsible for STEE’s pro-neurogenic effect; however, they were not acting alone but might have synergistic effects when combined.

### 2.2. Mono-CQAs in STEE Regulated Cyclin Expressions through p38 Activation in Differentiating hNSCs

**Figure 2:**
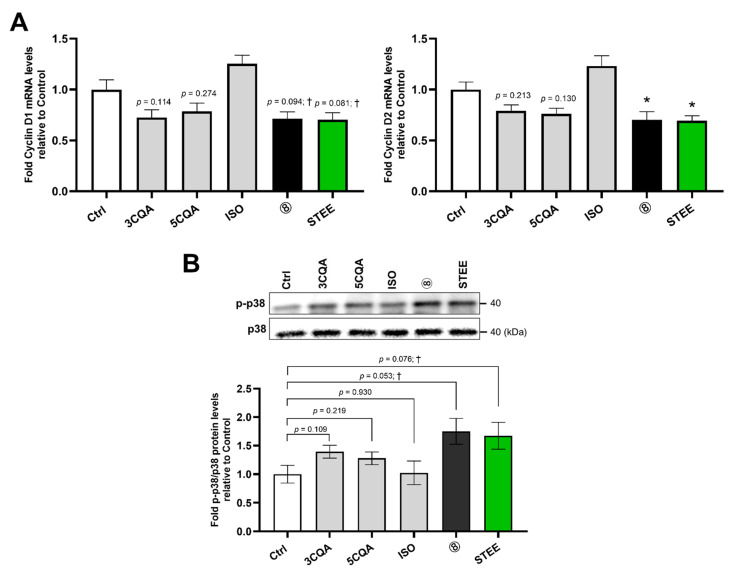
Mono-CQAs in STEE reduced Cyclin D mRNA expressions in hNSCs. (**A**) Cells were incubated with STEE and group 8’s compounds for 24 h, and Cyclin D1 and D2 mRNA (*CCND1* and *CCND2*) levels were measured by qRT-PCR. The relative values are the mean ± SEM (n = 3–4) compared with the control. One-way ANOVA with Dunnett’s post hoc test for the comparisons: * *p* < 0.05; † *p* < 0.1. (**B**) Cells were treated with STEE and group 8’s compounds for 60 min. The amounts of p-p38 and p38 were detected by immunoblotting, and band intensities were quantified. Data represent mean ± SEM (n = 3) when compared with control. Student’s *t*-test (unpaired) for the comparisons: † *p* < 0.1. A description of the number for the treatment group is indicated in Table 1.

In differentiating hNSCs, we further examined the effect of STEE and its compounds on the mRNA levels of Cyclin D1 and D2 (*CCND1* and *CCND2*), which are D-type cyclins positively regulating G1 to S phase cell cycle transition [23]. Both *CCND1* and *CCND2* decreased 1.3-fold in STEE, and group 8 compared to control groups; however, only the *CCND2* decrease reached statistical significance (*p* < 0.05) (Figure 2A). A single treatment with 3CQA or 5CQA also decreased *CCND1* and *CCND2* expressions (*CCND1*: approximately 1.25-fold; *CCND2*: approximately 1.23-fold), while ISO treatment slightly increased each transcript (Figure 2A). These results suggest that among the three compounds, which are suggested to contribute to STEE’s pro-neurogenic effect (Figure 1), 3CQA and 5CQA (mono-CQAs) regulated the cell cycle and inhibited G1 to S phase transition by suppressing cyclin D expression.

Next, we checked the effect of STEE and its compounds on the p38 activity by quantification of its phosphorylated active form using immunoblotting. The p38 is an upstream regulator of cell cycle progression and proliferation [24]. As shown in Figure 2B, treatment with STEE and group 8 increased the ratio of phosphorylated (p)-p38 to p-38 in hNSCs (*p* < 0.1, approximately 1.7-fold). The ratio of p-p38 was also increased after a single treatment with 3CQA or 5CQA (*p* = 0.109, 1.39-fold and *p* = 0.219, 1.27-fold, respectively), while no significant effect was observed in the ISO-treated hNSCs (Figure 2B).

Altogether, these findings suggest that mono-CQAs in STEE, 3CQA, and 5CQA might be responsible for G1 phase cell cycle arrest through the activation of p38, which might contribute to its pro-neurogenic effect.

### 2.3. ISO in STEE Regulated Differentiation-Related Transcriptional Factors and GSK3β Activity in Differentiating hNSCs

**Figure 3:**
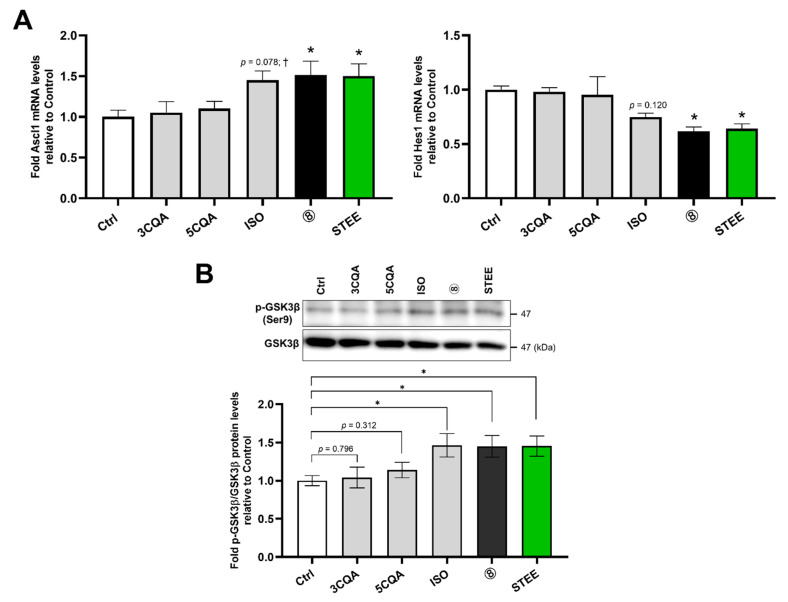
ISO in STEE regulated bHLH transcription factors expression in hNSCs. (**A**) Cells were treated with STEE and group 8’s compounds for 24 h. Ascl1 and Hes1 mRNA (*ASCL1* and *HES1*) levels were measured by qRT-PCR. The relative values are the mean ± SEM (n = 3–4) compared with the control. One-way ANOVA with Dunnett’s post hoc test for the comparisons: * *p* < 0.05; † *p* < 0.1. (**B**) Cells were treated with STEE and group 8’s compounds for 60 min. p-GSK3β and GSK3β expression were monitored by immunoblotting. The relative amounts are the mean ± SEM (n = 3) compared with the control. Student’s *t*-test (unpaired) for the comparisons: * *p* < 0.05. A description of the number for the treatment group is indicated in Table 1.

In differentiating hNSCs, we then investigated the effects of STEE and its compounds on the mRNA levels of Achaete-scute homolog 1 (Ascl1) and Hairy enhancers of split 1 (Hes1) (*ASCL1* and *HES1*), which are basic helix-loop-helix (bHLH) transcription factors regulating stem cell fate determination [25,26]. As shown in Figure 3A, treatment with STEE and group 8 significantly increased *ASCL1* (1.5-fold) and decreased *HES1* (1.4-fold) in hNSCs compared to control (*p* < 0.05 for all cases). Among the single compound treatments, *ASCL1* increased 1.45-fold (*p* = 0.078), and *HES1* decreased 1.25-fold (*p* = 0.120) in ISO-treated hNSCs (Figure 3A). The results suggest that ISO in STEE regulated transcription factors and directed differentiating NSCs fate toward neuronal.

Glycogen synthase kinase 3 β (GSK3β) is a serine/threonine kinase playing major roles in cell structure formation, cell division and differentiation, and apoptosis [27,28]. We examined the effects of STEE and its compounds on the activity of GSK3β by detecting its phosphorylated active form in western blot because ISO has been shown to act as a GSK3β inhibitor [22,29]. STEE, group 8, and ISO single treatment significantly increased the ratio of p-GSK3β to GSK3β (approximately 1.45-fold) in hNSCs (Figure 3B), suggesting ISO in STEE was the compound of interest that inhibited GSK3β activity in hNSCs.

Taken together, these data provide evidence that ISO in STEE directed the fate of differentiating NSCs toward neuronal lineage through regulating transcription factors expression and GSK3β activity.

### 2.4. STEE and Its Major Compounds Stimulated Mitochondrial Activity through PGC-1α Regulation in hNSCs-Derived Immature Astrocytes

**Figure 4:**
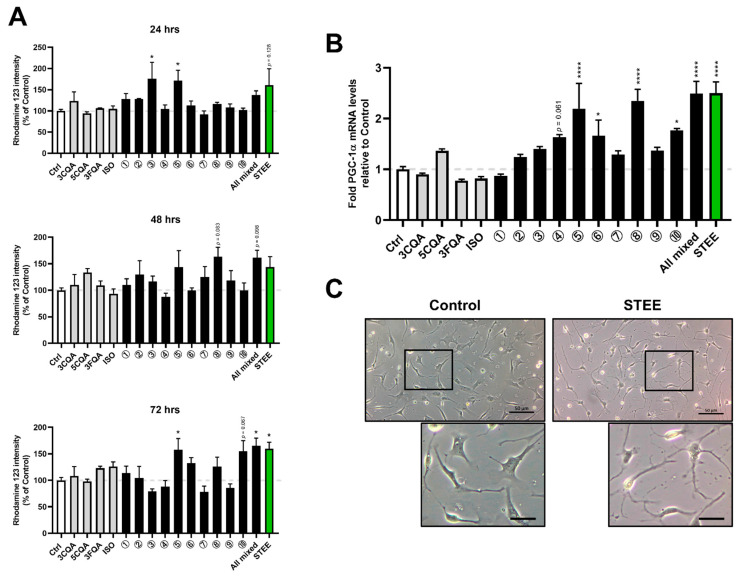
STEE and its major polyphenolic constituents stimulated the mitochondrial activity of hNSCs-derived immature astrocytes. (**A**) hNSCs were plated in a 96 well plate, induced differentiation into astrocytes, and pre-treated with STEE and its major constituents; 3CQA, 5CQA, 3FQA, or ISO, for the indicated times (24, 48, or 72 h). Cells were then further incubated with Rh123 for 20 min, and the fluorescence intensity generated by Rh123 was monitored at λex = 507 nm and λem = 529 nm. The percentages shown are the mean ± SEM (n = 3–6). One-way ANOVA with Dunnett’s post hoc test for the comparisons: * *p* < 0.05. (**B**) Cells were incubated with STEE and its constituents for 48 h, and PGC-1α mRNA (*PPARGC1*) expression was measured. The relative values are the mean ± SEM (n = 3) compared with the control. One-way ANOVA with Dunnett’s post hoc test for the comparisons: * *p* < 0.05, **** *p* < 0.0001. A description of the number for the treatment groups is indicated in Table 1. (**C**) Phase-contrast microphotographs of hNSC-derived astrocytes cultured for 48 h in the absence or presence of STEE (50 µg/mL). High magnification views of boxed areas are shown below panels. Scale bar = 50 µm in panels and 20 μm in insets.

Next, we investigated the effects of STEE and its compounds on astrocyte maturation and astrocytic mitochondrial biogenesis in hNSC-derived immature astrocytes. Mitochondrial biogenesis in astrocytes is a critical process controlling astrocyte maturation [30]. We measured Rhodamine 123 (Rh123) uptake by mitochondria of hNSCs-derived immature astrocytes. STEE or compounds were added to hNSC-derived immature astrocytes followed by measurement of Rh123 intensity (at 24, 48, and 72 h post-treatment). The treatment groups 3 and 5 significantly enhanced mitochondrial Rh123 fluorescence intensity 24 h post-treatment. The treatment group 8 and all four compounds (All mixed) showed an increasing trend of fluorescent intensity (*p* = 0.097 and *p* = 0.082, respectively) at 48 h. The treatment groups 5, 8, and STEE significantly enhanced fluorescent intensity after 72 h of treatment (Figure 4A). These results indicate that the compounds in STEE did not have much role in mitochondrial biogenesis when they were alone, but the combinations of 3CQA, 5CQA, and ISO contributed to mitochondrial activation of hNSC-derived immature astrocytes.

We next studied the effects of STEE and its compounds on mRNA levels of PGC1-α (*PPARGC1*), a master regulator of mitochondrial biogenesis [31], in hNSC-derived immature astrocytes. As shown in Figure 4B, *PPARGC1* levels in hNSC-derived immature astrocytes were significantly upregulated (*p* < 0.0001, approximately 2.5-fold) by STEE treatment for 48 h. Among the combinations, the treatment groups 5, 8, and All mixed significantly increased *PPARGC1* levels (*p* < 0.0001). Observation of hNSC-derived astrocytes following STEE treatment showed an increase in the number of astrocytes with branched processes (Figure 4C). This suggests that mitochondrial activation by STEE could increase astrocyte morphological complexity.

Altogether, these data indicate that the main compounds that contributed to the PGC-1α-mediated astrocytic mitochondrial activation by STEE were 3CQA, 5CQA, and ISO, which were not acting alone but in combination.

### 2.5. STEE and Its Compounds Regulated the Activity of p38 and GSK3β in hNSCs-Derived Immature Astrocytes

**Figure 5:**
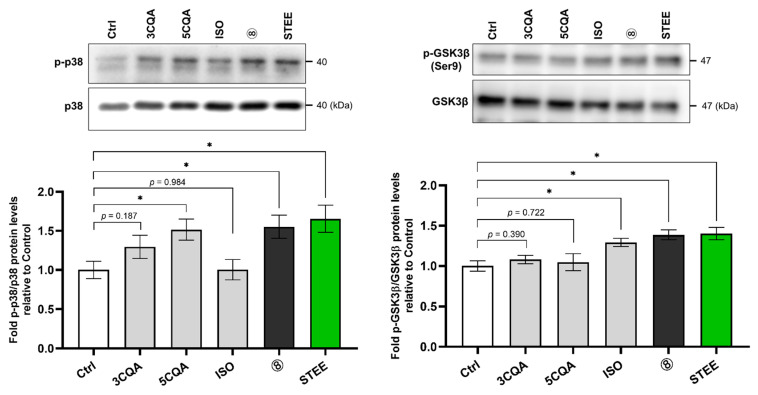
Polyphenolic constituents in STEE regulated p38 and GSK3β activity in hNSC-derived immature astrocytes. Cells were treated with STEE and group 8’s compounds for 60 min. The amounts of p38, GSK3β, p-p38, and p-GSK3β expression in each cell lysate were detected by immunoblotting and quantified. The relative amounts are the mean ± SEM (n = 3) compared with the control. Student’s *t*-test for the comparisons: * *p* < 0.05. A description of the number for the treatment group is indicated in Table 1.

As mono-CQAs regulated p38 activity and ISO regulated GSK3β activity in hNSCs, we further investigated their effects in hNSCs-derived immature astrocytes using immunoblotting. As shown in Figure 5, exposure to STEE itself and group 8 for 60 min significantly increased the ratio of p-p38 to p38 (approximately 1.6-fold) and p-GSK3β to GSK3β (approximately 1.4-fold) compared to the control group (*p* < 0.05 in all cases) in hNSC-derived immature astrocytes. Immature astrocytes were also exposed to 3CQA, 5CQA, 3FQA, and ISO independently for 60 min. The ratio of p-p38 to p38 was increased 1.51-fold by 5CQA (*p* < 0.01) and 1.29-fold by 3CQA (*p* = 0.187) compared to control. The ratio of p-GSK3β to GSK3β significantly increased 1.29-fold by ISO compared to the control (Figure 5). 3FQA treatment did not affect the phosphorylation levels of both proteins (Figure S3).

These data suggest that mono-CQAs could regulate p38, and ISO could regulate GSK3β activity in hNSC-derived immature astrocytes also.

### 2.6. Introduce of BBB-like Structure Abolished the Effect of STEE and Its Compounds on the Neuronal Marker Expression in Differentiating hNSCs

Finally, we investigated the BBB permeability of the compounds. We used an established BBB model kit that well mimics in vivo BBB features and has been used in previous studies to investigate the BBB’s permeability to plant-based molecules [32,33,34]. Treatment with STEE (50, 100, 200 µg/mL) via BBB-like structure did not significantly affect the *TUBB3* levels of differentiating hNSCs compared to the control (without treatment) (Figure S2). Moreover, no significant change in *TUBB3* expression was observed when the hNSCs were treated with a mixture of the four compounds via the BBB kit insert (Figure S2). STEE treatment without BBB kit inserts in the same time course upregulated *TUBB3* expression predominantly compared to control (*p* < 0.01), suggesting that the compounds which are responsible for STEE’s pro-neurogenic effect barely penetrated the BBB.

## 3. Discussion

**Figure 6:**
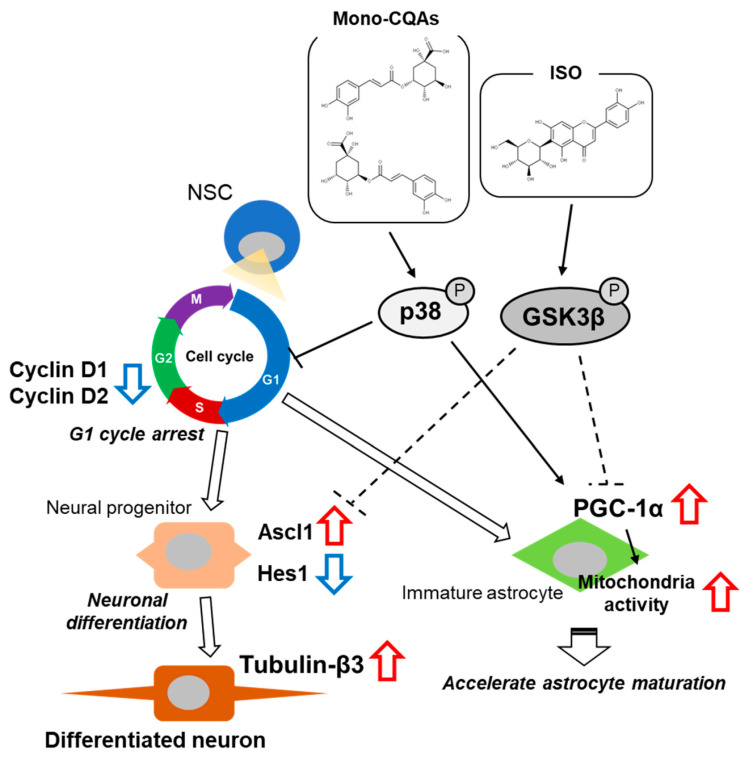
Schematic diagram of the effect of STEE’s major polyphenolic constituents on hNSCs neuronal differentiation and immature astrocyte mitochondrial activity. CQAs triggers p38 activation and induce G1 cycle arrest of NSCs, thus increasing the potential for cell differentiation. In turn, ISO favors neuronal cell fate decisions by inactivating GSK3β and inducing a regulation of transcription factors (upregulation of *Ascl1* and downregulation of *Hes1*). These effects synergistically cause neuronal differentiation. Activation of p38 and inactivation of GSK3β also promote *PGC-1α* expression in immature astrocytes. p38: p38 mitogen-activated protein kinase; GSK3β: glycogen synthase kinase 3β; Ascl1: achaete-scute homolog 1; Hes1: hairy and enhancer of split 1; PGC-1α: peroxisome proliferators-activated receptor-γ co-activator-1α.

In the present study, we have identified the bioactive compounds of interest in STEE by investigating the affected molecular targets of the compounds. We have shown that each of the compounds acts synergistically and co-regulated the cell cycle transition and the cell fate determination and contributes to STEE’s neuronal differentiation-promoting effects in hNSCs. We have also found that STEE and its compounds stimulate mitochondrial activity in immature astrocytes through PGC-1α enhancement (Figure 6).

In recent years, technological advances in screening, isolation, characterization, and optimization have reinvigorated plant extract-based drug discovery. Plant extracts, either standardized or pure compounds, provide new drug leads; however, they are not without challenges. Plant extracts consist of multicomponent mixtures. It has long been argued that unmodified plant extracts may have suboptimal efficacy or ADMET properties (i.e., absorption, distribution, metabolism, excretion, and toxicity), requiring chemical modification to standardize their functional components [11,35]. On the other hand, some are interested in discovering the main drug-like pure compounds in plant extracts. However, sometimes several bioactive compounds act synergistically and contribute to the ultimate beneficial effect [10,36,37]. Therefore, the pure compound identification strategy very commonly ends in poor reproducibility and translatability [10]. Therefore, identifying the bioactive compounds of interest in a plant extract and understanding their targeted pathways responsible for the observed health benefits need to be well understood to exploit their full potential [38]. In our previous study, we reported that STEE exerts beneficial effects on aging-related cognitive health by promoting neuronal differentiation and neuronal energy metabolism and extending the length of the astrocytic process in vitro [19]. We have identified four major polyphenols rich in STEE. We, therefore, conducted the present study with the aim of identifying the bioactive polyphenol responsible for STEE’s neuroprotective effects.

Tubulin-β3 is a component of stabilized neuronal microtubules and is well known specific marker of neurons. Both the upregulation and the post-translational processing of tubulin-β3 are essential for neuronal differentiation [39]. Moreover, neuron-specific expression of tubulin-β3 begins during or before the final division of neuronal progenitor cells [40]. Therefore, the hypothesis that the expression of tubulin represents an early molecular event in neuronal differentiation has long been supported, and thus the results in this study may well reflect the pro-neurogenic potential of the extract and its compounds.

There is a tight relationship between cell cycle progression and stem cell lineage commitment, which is crucial for stem cell fate [41,42]. In particular, the early G1 phase of the cell cycle plays an important role in stem cell differentiation. In most somatic cells, including NSCs, progression from G1 to S phase requires the mitogen-dependent activity of cyclins, namely the cyclin-dependent kinase 4 (CDK4)-cyclin D complex. Moreover, a longer cell cycle has been observed during NSC differentiation [43]. In fact, it has been reported that the G1 phase of adult NSCs doubles during the induction of differentiation, and inhibition of CDK4-cyclin D increases the percentage of cells in the G1 phase and promotes differentiation [44]. Moreover, several reports have suggested that p38, a class of mitogen-activated protein kinases (MAPKs), negatively regulates cyclin D expression and associated cell cycle responses [45,46,47]. CQA derivatives have been reported to regulate cell cycle arrest-related signals in stem cells and also promote the differentiation of hNSCs into lineage cells by negatively regulating the G1/S transition and increasing G0/G1 arrest [48,49]. In this study, mono-CQAs decreased cyclin D expressions and increased the phosphorylated form of p38 in differentiating hNSCs, suggesting induced G1 arrest by suppressed cyclin D through p38 activation. However, single or co-treatment with mono-CQAs did not sufficiently increase the transcript levels of tubulin-β3. This suggests that mono-CQAs in STEE may play a role in providing NSCs with a chance for lineage differentiation by inducing G1 arrest.

It is well known that the fate of NSCs, i.e., their proliferation and differentiation, is controlled by the expression dynamics of bHLH transcription factors [25,26]. In particular, activator-type bHLH factors such as Ascl1 show a sustained increase during NSCs neuronal differentiation, while the expression of repressor-type of bHLH factors such as Hes1 is suppressed. Moreover, several reports have shown that GSK3β, a serine/threonine protein kinase and a component of the Wnt/β-catenin signaling cascade, acts as a negative regulator in the proliferation and differentiation of NSCs [50,51]. It has been reported that the blockade of β-catenin nuclear translocation via active GSK3β decreases pro-neural bHLH factor expressions [52,53]. GSK3β substrates include transcription factors and metabolic regulators, and some bHLH factor family members, such as Ascl1, have been reported to own specific interaction sites with phosphorylated GSK3β [54], suggesting the possibility of regulation of NSCs proliferation and differentiation by crosstalk between the activity of this kinase and transcription factors. In this study, ISO in STEE regulated the expression of transcription factors toward predominately neuronal differentiation and increased inactivated GSK3β. Our findings are in line with the previous studies that have reported that ISO attenuates neuronal damage by directly acting on GSK3β and inhibiting its activity [22,29]. However, single ISO treatment, as well as mono-CQAs, did not significantly affect the expression of neuronal markers. Therefore, it suggests that when in combination, ISO and mono-CQAs interact favorably and complement each other to accelerate STEE’s neuronal differentiation-inducing effects.

Astrocyte maturation is an important developmental process for the normal functioning of the CNS, and this maturation is characterized by morphological parameters [55]. Matured astrocytes elaborate their leaflet processes and infiltrate the neuropil. This astrocytic process is an important functional structure and facilitates intercellular communication and enwrapping of synapses and contributes to neurotransmitter uptake and synapse development and stabilization [56,57]. It is not surprising that disrupted physical contact between astrocytes and synapses contributes to the etiology and progression of neurological and psychiatric disorders. For example, altered astrocytic glutamate uptake and associated neuronal excitotoxicity are associated with several neurodegenerative diseases [58]. Moreover, astrocytes in the early onset of neurodegenerations have been reported to show morphological deficits characterized by decreased fine processes and territory volume [9]. The mechanisms that regulate the morphological maturation of astrocytes are poorly understood [59] but maintaining astrocyte morphological integrity may represent a promising therapeutic target.

A recent study has indicated that the astrocytic mitochondrial biogenesis and consequent metabolic shift to oxidative phosphorylation, and transient activation of their master regulator PGC-1α, are critically important for the morphological development of astrocytes [30]. Therefore, in this study, the PGC-1α activity in astrocytes, shortly after differentiation from hNSCs, was chosen as an indicator of immature astrocyte morphogenic potential. We found that coordinated action of mono-CQAs (here, significantly by 5CQA) and ISO significantly upregulated PGC-1α expression in hNSC-derived immature astrocytes. Although PGC-1α is regulated by several factors [60], such as cAMP response element-binding protein (CREB) [61] or mammalian target of rapamycin (mTOR) [62], a model has also been proposed where p38 and GSK3β could be downstream regulators of a complex activation pathway [63,64]. Since 5CQA regulated p38 activity and ISO regulated GSK3β activity in hNSC-derived immature astrocytes also, it is possible that the regulation of kinases activity by each compound synergistically contributed to the promotion of PGC-1α transcription.

Single 3FQA treatment did not show significant effects in the study but subtly increased Rh123 intensity and PGC-1α expression. A previous study has reported that ferulic acid, a metabolite of FQA, improves the energy metabolism of the rodent brain [65], suggesting 3FQA may not be completely unrelated to the modulation of astrocytic energy metabolism.

The bioactivity of STEE and its compounds on NSCs differentiation is discussed above; however, the results also indicate that STEE compounds were impermeable to the BBB. Although the BBB kit predicts well the permeability of molecules into the brain, it may not represent the in vivo microenvironment entirely. Adding to this point, a previous mass spectrometric study by Su et al. demonstrated that mono-CQAs could pass through the BBB and are detectable in the brain in vivo [66]. Therefore, the compound’s accumulative capacity upon continuous exposure and its ability to transport into the brain parenchyma should be carefully considered. Alternatively, some previous studies indicate that the median eminence (ME), which is adjacent to the arcuate nucleus (Arc) and is a so-called circumventricular organ, has access to circulating molecules because of the lack of a typical BBB and its permeable vasculatures [67,68]. The Arc, located at the base of the hypothalamus, contains neurons that sense circulating hormones and nutrients, so the Arc-ME has been considered a primary site of exchange between the systemic circulation and the brain [69]. Interestingly, continuous neurogenesis has also been detected in the ME and Arc of the adult human brain [70,71]. Although the mechanisms underlying the circulating molecule’s entry into the Arc are not fully understood, given the previous finding that STEE stimulated hippocampal neurogenesis in vivo [19], we hypothesize that STEE compounds would input signals through Arc-ME.

The present study provides the first comprehensive in vitro assessment of pro neurogenic effects of major polyphenols of STEE. We have found that the STEE, rather than a single isolated compound, stimulated neuronal differentiation of hNSCs. Our previous study showed that the crude STEE could ameliorate age-associated cognitive decline and induce neurogenesis in vivo [19]. While the neurobeneficial effects of plant extracts and their polyphenols are well documented, the poor bioavailability of phenolic compounds remains the major challenge for their successful clinical translation. Previous clinical studies reported that CQAs and ISO, despite their low absorption rate, could be detected in plasma unchanged after oral intake at nutritionally relevant doses [72,73]. Therefore, it is reasonable to assume that the synergistic bioactivity of CQAs and ISO in STEE would also be observed in the in vivo and clinical studies. However, further research is required to confirm the human bioavailability and metabolism of STEE and its polyphenols.

In conclusion, the findings in this study strongly support the idea that polyphenolic compounds in the plant extracts can accelerate NSCs differentiation and immature astrocyte development, not by acting individually, but through their synergistic effects generated by each bioactive substance. Although further experiments will be necessary to elucidate the underlying mechanisms, these results reinforce the notion that STEE stimulates the development of hNSCs in vitro culture mainly through the regulation of p38 and GSK3β.

## 4. Materials and Methods

### 4.1. Preparation of STEE and Chemical Reagents

STEE was prepared as previously reported [19]. In brief, the plants were extracted in an automatic extractor (E-916, Buchi AG, Uster, Switzerland) with ethanol/water (80:20, *v*/*v*), the solvent was removed via rotary evaporation/freeze-drying, and re-suspended in ethanol/water (70:30, *v*/*v*) at 100 mg/mL. The plant material was donated by Nippo Co., Ltd. (Osaka, Japan).

Four compounds, 3CQA, 5CQA, 3FQA, and ISO, which were found to be rich in STEE in our previous study, were commercially purchased and used in the experiments. Pure chemicals of 3CQA (≧98%), 5CQA (≧99%), and 3FQA (≧98%) were purchased from Nagara Science (Gifu, Japan), and ISO (≧98%) were purchased from Sigma-Aldrich (St. Louis, MO, USA). The compounds were suspended in ethanol/water (70:30, *v*/*v*) at 25 or 50 mM, divided into small aliquots, and stored at −80 °C until used. The structures of the compounds are shown in Figure S1.

### 4.2. Cells and Cell Culture

Primary hNSCs (HS820-20f, Cell Applications, San Diego, CA, USA) were cultured as previously reported [19]. The cells were maintained in the proliferation medium as free-floating aggregates (Neurospheres) at 37 °C in a humidified incubator with 95% air/5% CO_2_. Neurospheres were dissociated using StemPro Accutase reagent (Gibco-Thermo Fisher, Grand Island, NY, USA) and passaged when the diameter of the spheres reached a maximum of 300 μm (every 10–12 days before the cells started unexpected differentiation). The proliferation medium consisted of Knockout Dulbecco’s modified Eagle’s medium (DMEM)/F12 (1:1, *v*/*v*), 2% StemPro Neural Supplement, 20 ng/mL basic fibroblast growth factor (bFGF), 20 ng/mL epidermal growth factor (EGF), 2 mM Glutamax Supplement, and 1% anti-bacterial penicillin/streptomycin.

### 4.3. hNSC Differentiation

In order to induce hNSCs differentiation into the neural lineages, the cells were cultured as adherent. The maintained neurospheres were dissociated with Accutase reagent (Gibco), plated on culture vessels (6-, 24-, or 96-well plates) pre-coated with poly-D-lysine (PDL; Corning, Corning, NY, USA) and Cultrex laminin (R&D Systems, Minneapolis, MN, USA), and cultured in neural or astroglial differentiation medium for differentiation into neurons or astrocytes. The neural differentiation medium and the proliferation medium had the same components, except the latter one had growth factors. The astroglial differentiation medium consisted of DMEM, 1% N-2 supplement (Gibco), 1% fetal bovine serum (FBS; Gibco), and 1% penicillin/streptomycin. After the differentiation induced by the medium with treatment samples, each culture was used for subsequent experiments.

### 4.4. Treatment of STEE and Its Compounds to hNSCs

In this study, the treatment concentration of STEE to the cells was set at 50 µg/mL unless otherwise indicated, which was optimal for the induction of hNSCs differentiation in the previous study [19]. The treatment concentrations of each chemical compound in the culture were set as follows: 3CQA = 0.50 µM, 5CQA = 0.70 µM, 3FQA = 0.85 µM, ISO = 0.48 µM. These concentrations were equivalent to that contained in 50 µg/mL of STEE and were calculated from the quantitative results of the chemical analysis [19]. We treated the cells with STEE and its polyphenols either independently or in combination. The combinations of the compounds are explained in Table 1.

hNSCs were seeded onto PDL/laminin-coated culture vessels (10,000 cells/cm^2^), stabilized in proliferation medium for 48 h, and incubated for 60 min or 24 h with the replaced neural differentiation medium containing the extract or compounds. Cells incubated for 24 h were subjected to RNA extraction for reverse transcription, and cells incubated for 60 min were subjected to protein extraction for immunoblotting.

### 4.5. Treatment of STEE and Its Compounds to hNSC-Derived Immature Astrocytes

Also, hNSCs were seeded onto PDL/laminin-coated culture vessels (10,000 cells/cm^2^), stabilized in proliferation medium for 48 h, and incubated for another 48 h with replaced astroglial differentiation medium to differentiate cells into astrocytes. Cells were pre-treated with the extract or its compounds, and their mitochondrial activity was measured at 24, 48, and 72 h post-treatment. Cells were also incubated with the samples for 48 h or 60 min for the gene and protein expression analysis.

### 4.6. Assessment of BBB Permeability

A BBB in vitro model kit (MBT-24, PharmaCo-Cell, Nagasaki, Japan), which consists of primary cultures of brain capillary endothelial cells, pericytes, and astrocytes, was used to assess the BBB permeability of four major polyphenolic compounds in STEE. Cells comprising the kit were cultured for 5 days according to the manufacturer’s protocol. Transendothelial electrical resistance (TEER) was confirmed to be higher than 150× cm^2^ using Millicell ERS-2 (Millipore, Billerica, MA, USA) for the manifestation of BBB-like function, and the insert of the kit, which consist of vascular endothelial cells and pericytes, were used in following experiments.

The culture medium of hNSCs stabilized for 48 h in a 24-well plate was replaced with a neural differentiation medium, and the established BBB kit insert was immediately put onto the wells. In the adult mammalian brain, NSCs are maintained in the neurovascular niche, and the transwell system has been used as the 2D model for understanding the reciprocal cellular mechanisms (including BBB properties) of NSCs and brain endothelial cells [74,75]. Subsequently, STEE (50, 100, or 200 µg/mL) or a mixture of four polyphenols (equivalent to a concentration in 50 µg/mL of the extract) were added to the insert, i.e., the luminal side, and cells were incubated for another 24 h. The cell cultures without the BBB kit insert were also incubated with STEE (50 µg/mL) to make positive controls. After the incubation, cells were subjected to RNA extraction.

### 4.7. Measurement of Intracellular Mitochondrial Activity

Intracellular mitochondrial activity was measured in the assay using Rh123. Rh123 is readily taken up by intracellular mitochondria, and mitochondrial activity can be evaluated by measuring its fluorescence intensity [76]. hNSC-derived immature astrocytes were pre-treated with the extract or its compounds for 24–72 h, washed with PBS, and then incubated with 10 µg/mL of Rh123 solution for 20 min at 37 °C. Cells were washed again and lysed with 1% Triton-X solution for 30 min at room temperature in the dark. Cell lysates were transferred into a black clear-bottom 96-well plate, then the fluorescence intensity was measured at λex = 507 nm and λem = 529 nm with a plate reader (Varioskan LUX, Thermo Fisher Scientific, Rockford, IL, USA).

### 4.8. RNA Isolation and qRT-PCR

Total RNA was isolated from the harvested cells using the RNeasy mini kit or micro kit (Qiagen, Venlo, The Netherlands), and cDNAs were prepared using the SuperScript IV VILO kit (Applied Biosystems, Foster City, CA, USA) according to the manufacturer’s protocols.

Quantitative real-time polymerase chain reaction (qRT-PCR) was performed using TaqMan Gene Expression Master Mix and TaqMan Primer in a 7500 Fast Real-Time PCR System (All are from Applied Biosystems) as previously reported [19]. The following TaqMan primers for RT-PCR were used: *GAPDH* (Hs02786624_g1), *TUBB3* (Hs00801390_s1), *ASCL1* (Hs00269932_m1), *HES1* (Hs00172878_m1), *CCND1* (Hs00765553_m1), *CCND2* (Hs00153380_m1), *PPARGC1* (Hs00173304_m1). *GAPDH* levels were used as an internal control to determine the relative expression levels of each transcript.

### 4.9. Protein Extraction and Immunoblotting

Total proteins of harvested cells were collected using ice-cold RIPA buffer (Sigma-Aldrich, St. Louis, MO, USA), supplemented with 1% protease inhibitor cocktail (Sigma-Aldrich) and 1% phosphatase inhibitor cocktail (nacalai tesque, Kyoto, Japan). Cells were incubated with the buffer on ice for 15 min, followed by ultrasonication. Lysates were subsequently centrifuged for 10 min at 16,000× *g*. The total protein concentration of samples was determined by Thermo Scientific’s Peirce BCA assay.

Equal amounts of protein per sample (5 or 10 µg) were applied to sodium dodecyl sulfate-polyacrylamide gel electrophoresis (SDS-PAGE). The electrophoresis was performed on 8–10% gels at 100 V. The separated proteins were blotted onto the PVDF membrane (Millipore). The membrane was blocked with 5% Bovine Serum Albumin (BSA) in Tris Buffered Saline with 0.1% Tween 20 (TBS-T), i.e., blocking buffer, for 30 min at room temperature. Then the proteins on the membrane were probed with primary antibodies diluted in blocking buffer overnight at 4 ºC. Three TBS-T washes preceded and followed by incubation with appropriate horseradish peroxidase (HRP)-linked secondary antibodies (Cell Signaling, Danvers, MA, USA) diluted in blocking buffer for 1 h at room temperature. Immunoreactive protein bands were visualized on Odyssey Fc Imaging System (LI-COR, Lincoln, NE, USA) with Thermo Scientific’s Pierce HRP substrate for chemiluminescence. The intensities of the detected band were quantified using ImageJ (NIH, Bethesda, MD, USA). The following primary antibodies were used: rabbit p38 MAPK (1:1000, Cell Signaling), rabbit p-p38 MAPK (1:1000, Cell Signaling), mouse GSK3α/β (1:1000, Santa Cruz, Dallas, TX, USA), mouse p-GSK3β (serine 9 (Ser9) phosphorylated, 1:1000, Santa Cruz).

### 4.10. Statistical Analysis

Data are expressed as means ± standard error of means (SEM). Normal distribution was tested by the Shapiro-Wilk normality test. A one-way analysis of variance (ANOVA) followed by Dunnett’s post hoc test was applied to compare each of the treatment conditions with the control group. For analyses of immunoblotting, Student’s *t*-test (unpaired) was used to compare the differences between the two groups. The level of significance was set at α < 0.05. All statistical analyses were performed using GraphPad Prism 8 (GraphPad) software.

## Figures and Tables

**Table 1:** The treatment group of combinations in this study.

Number of Compounds	Description	Treatment Group No.
Two	3CQA + 5CQA	1
	3CQA + 3FQA	2
	3CQA + ISO	3
	5CQA + 3FQA	4
	5CQA + ISO	5
	3FQA + ISO	6
Three	3CQA + 5CQA + 3FQA	7
	3CQA + 5CQA + ISO	8
	3CQA + 3FQA + ISO	9
	5CQA + 3FQA + ISO	10

## Data Availability

All data generated or analyzed during this study are included in this article Supplementary Materials.

## Supplementary Materials

The following are available online at https://www.mdpi.com/article/10.3390/ijms232315120/s1, Figure S1: Structures of the compounds used in this study; Figure S2: Expression of neuronal differentiation marker in hNSCs after the addition of samples across the BBB-like structure. STEE or polyphenols mixture was added to the established BBB kit inserts attached to hNSCs culture vessels. After 24 h of incubation, cells were subjected to RNA extraction and subsequent qRT-PCR for measurement of tubulin-β3 mRNA (*TUBB3*) levels. The values represent the mean ± SEM (n = 3). One-way ANOVA followed by Dunnett’s post hoc test for comparisons: ** *p* < 0.01; Figure S3: Treatment with 3FQA did not affect the phosphorylation levels of p38 and GSK3β in hNSC-derived immature astrocytes. Cells treated with 3FQA for 60 min were subjected to protein extraction and subsequent immunoblotting. The relative protein levels are the mean ± SEM (n = 3) compared with the control. Student’s *t*-test for the comparisons.
