# Supplementary material for: Interactions between Major Bioactive Polyphenols of Sugarcane Top: Effects on Human Neural Stem Cell Differentiation and Astrocytic Maturation
Source: Int J Mol Sci. 2022 Dec 1;23(23):15120. doi: 10.3390/ijms232315120 (PMC9738893; doi:10.3390/ijms232315120)
Supplement: Supplementary file 1 [file ijms-23-15120-s001.zip › Supplemental figures_revised.pptx]

## Slide 1
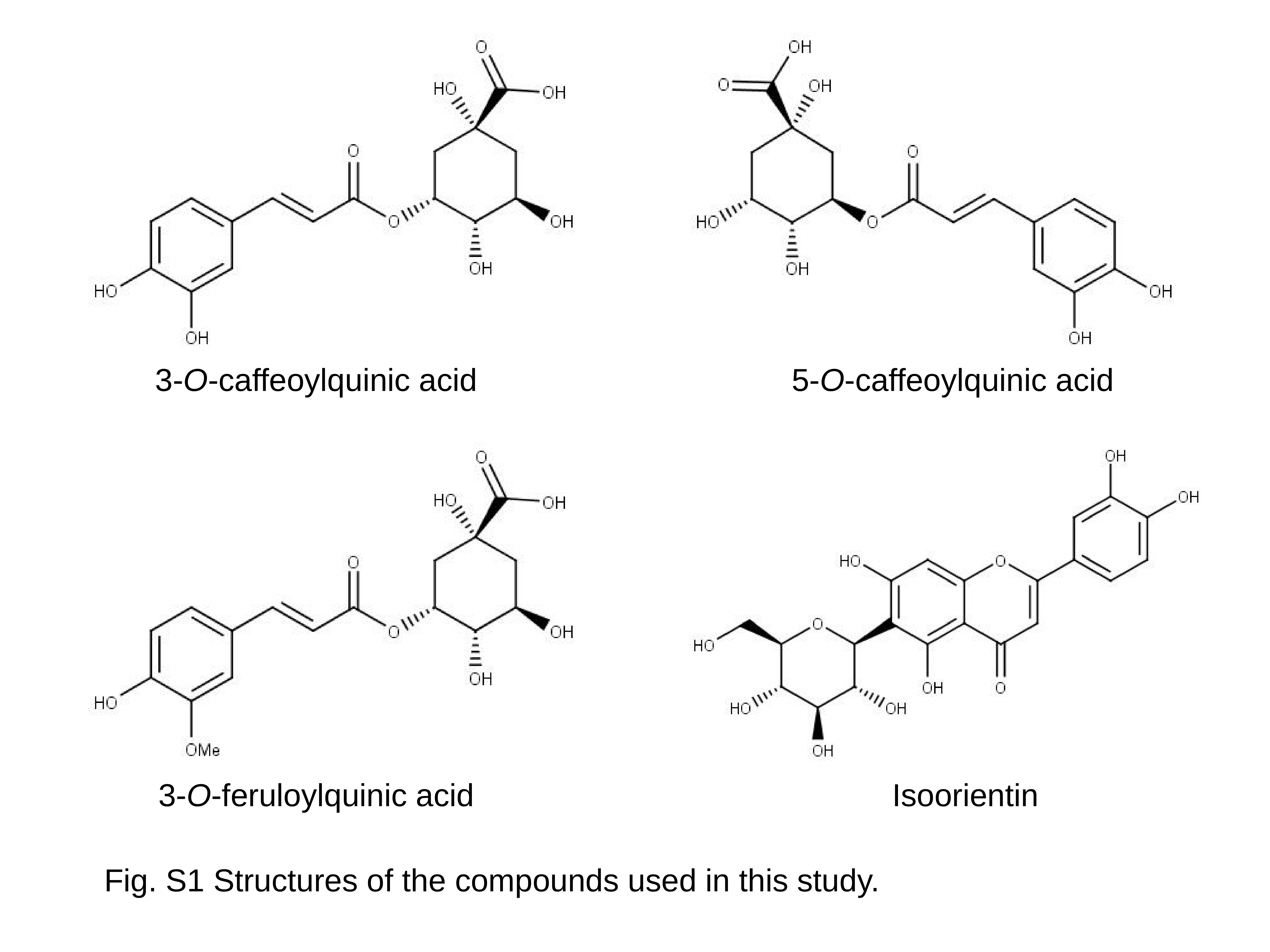

3-O-caffeoylquinic acid
5-O-caffeoylquinic acid
Isoorientin
3-O-feruloylquinic acid
Fig. S1 Structures of the compounds used in this study.

## Slide 2
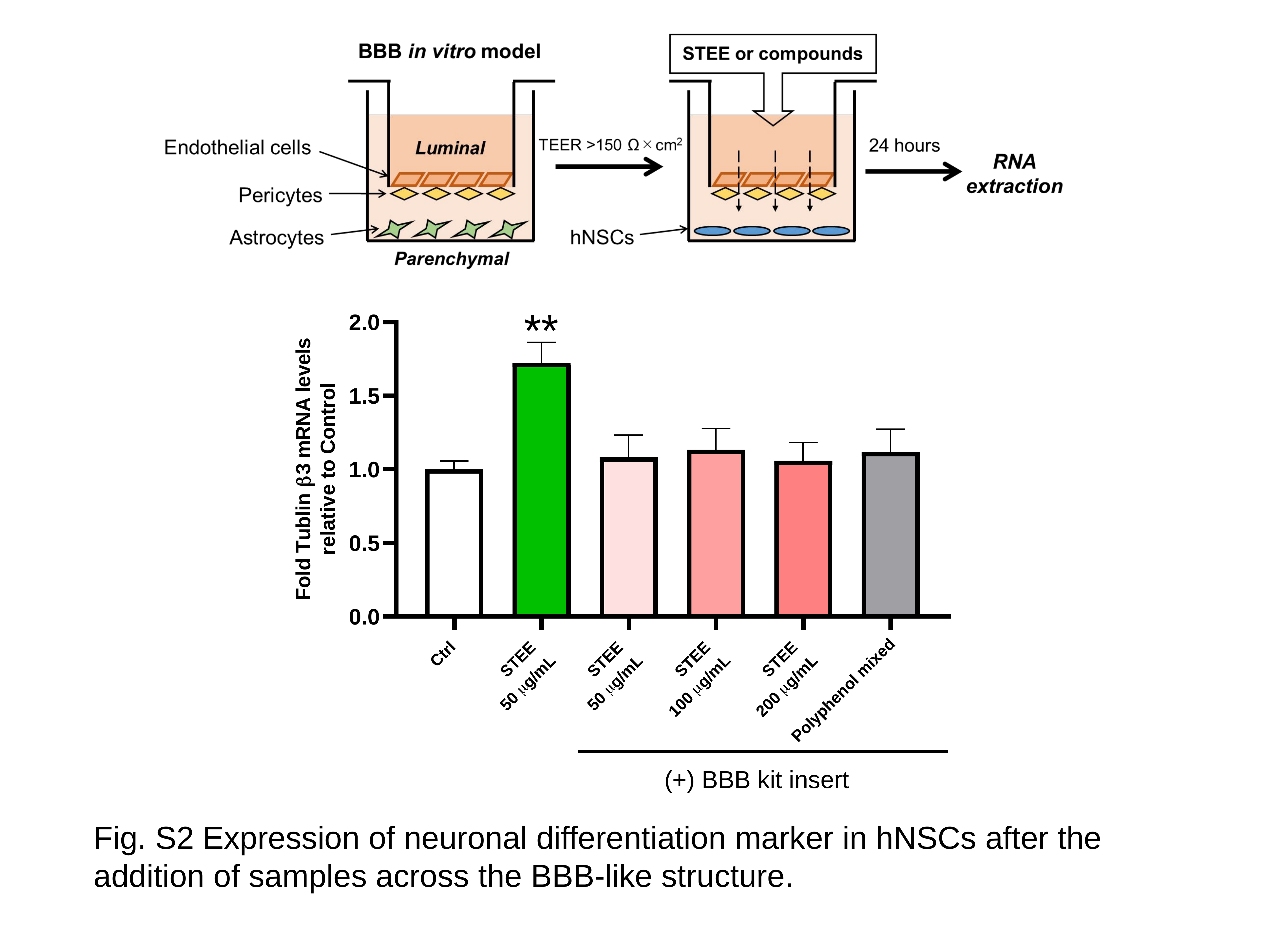

Fig. S2 Expression of neuronal differentiation marker in hNSCs after the addition of samples across the BBB-like structure.

## Slide 3
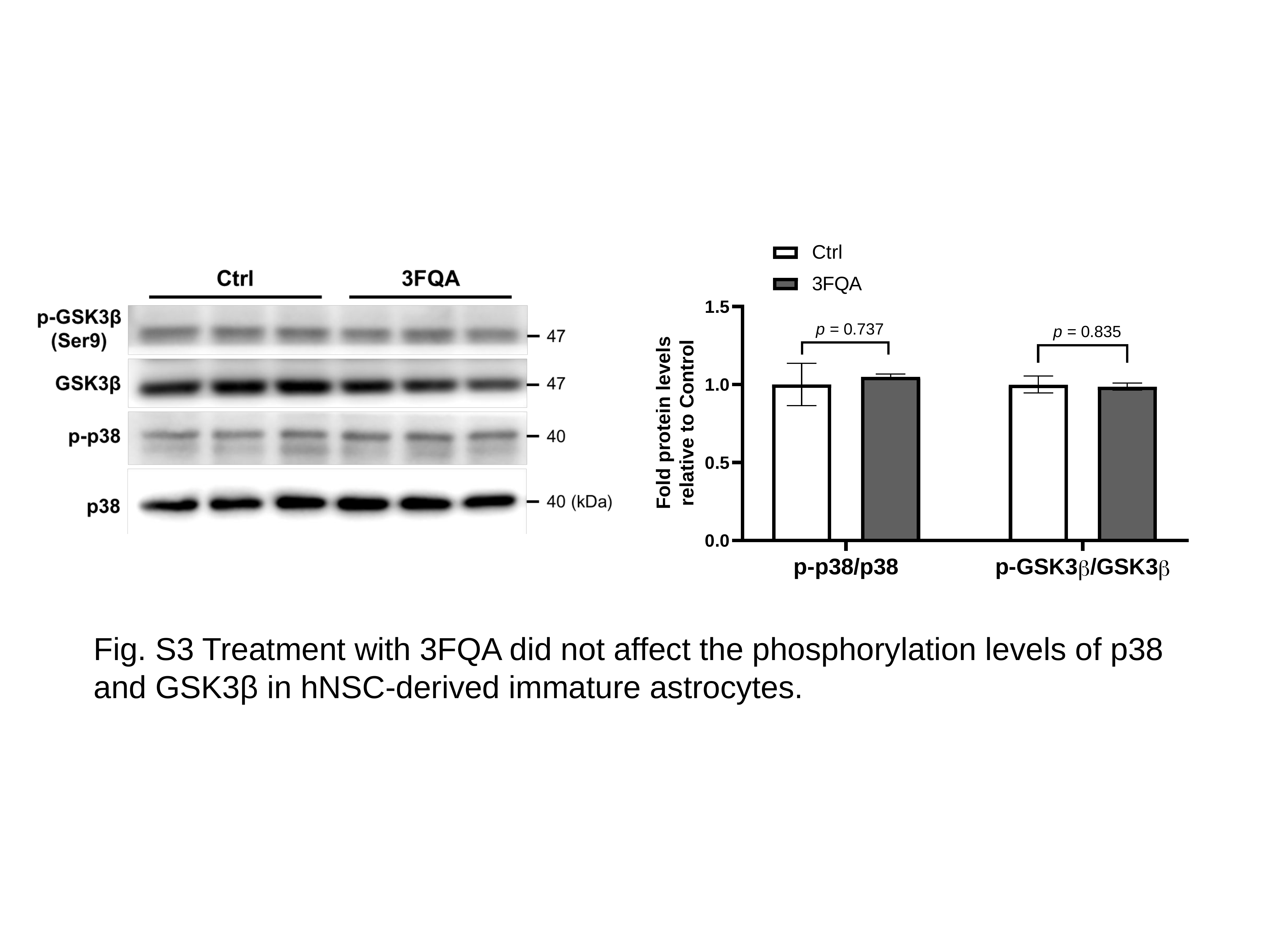

Fig. S3 Treatment with 3FQA did not affect the phosphorylation levels of p38 and GSK3β in hNSC-derived immature astrocytes.
